# Standard Chemoradiation and Conventional Brachytherapy for Locally Advanced Cervical Cancer: Is It Still Applicable in the Era of Magnetic Resonance–Based Brachytherapy?

**DOI:** 10.1200/JGO.18.00028

**Published:** 2018-06-21

**Authors:** Prachi Mittal, Supriya Chopra, Sidharth Pant, Umesh Mahantshetty, Reena Engineer, Jaya Ghosh, Sudeep Gupta, Yogesh Ghadi, Siji Menachery, Jamema Swamidas, Lavanya Gurram, Shyam Kishore Shrivastava

**Affiliations:** **All authors:** Tata Memorial Centre, Navi Mumbai, India.

## Abstract

**Purpose:**

Recent guidelines recommend magnetic resonance imaging–based brachytherapy (MRBT) for locally advanced cervical cancer. However, its implementation is challenging within the developing world. This article reports the outcomes of patients with locally advanced cervical cancer treated with chemoradiation and point A–based brachytherapy (BT) using x-ray– or computed tomography–based planning.

**Methods:**

Patients treated between January 2014 and December 2015 were included. Patients underwent x-ray– or computed tomography–based BT planning with an aim to deliver equivalent doses in 2 Gy (EQD2) > 84 Gy_10_ to point A while minimizing maximum dose received by rectum or bladder to a point or 2 cc volume to < 75 Gy EQD2 and < 90 Gy EQD2, respectively_._ The impact of known prognostic factors was evaluated.

**Results:**

A total of 339 patients were evaluated. Median age was 52 (32 to 81) years; 52% of patients had stage IB2 to IIB and 48% had stage III to IVA disease. There was 85% compliance with chemoradiation, and 87% of patients received four or more cycles. Median point A dose was 84 (64.8 to 89.7) Gy. The median rectal and bladder doses were 73.5 (69.6 to 78.4) Gy_3_ and 83 (73.2 to 90.0) Gy_3_, respectively. At a median follow-up of 28 (4 to 45) months, the 3-year local, disease-free, and overall survival for stage IB to IIB disease was 94.1%, 83.3%, and 82.7%, respectively. The corresponding rates for stage III to IVA were 85.1%, 60.7%, and 69.6%. Grade III to IV proctitis and cystitis were observed in 4.7% and 0% of patients, respectively.

**Conclusion:**

This audit demonstrates good 3-year outcomes that are comparable to published MRBT series. Conventional BT with selective use of interstitial needles and MRBT should continue as standard procedures until level-I evidence for MRBT becomes available.

## INTRODUCTION

Cervical cancer is one of the most common cancers in women in developing countries, with persistently high mortality due to advanced stage at presentation.^1^ For locally advanced cervical cancer (LACC), concurrent chemoradiation (CRT) and brachytherapy (BT) represent standard treatment.^2^ The addition of concurrent chemotherapy was associated with at least an 8% incremental gain in disease-free survival (DFS) in stage IIIB disease in a phase III randomized trial. Recently, results of two phase III randomized trials from our institution that used CRT with up to 70 to 75 Gy equivalent doses in 2 Gy (EQD2) to point A showed a 5-year DFS of 76.7% and 51.5% in patients with stage IB to IIB and IIIB disease, respectively.^3,4^ There is growing evidence from many mono-institutional experiences and the international External Beam Radiotherapy and MRI Based Image Guided Adaptive Brachytherapy in Locally Advanced Cervical Cancer (EMBRACE) study group indicating better outcomes with image-guided adaptive BT. A recent study from our institution that compared the EMBRACE study cohort with a historical cohort also demonstrated that there may be a gain in DFS with integration of magnetic resonance imaging (MRI)–based BT, and the cost investment of transition toward MRI-based BT may be justified in view of the gain in working life-years of the cured women.^5^ However, for both these studies, the comparison cohort was the historical cohort treated with BT at doses of 70 to 75 Gy delivered to point A.

Although most of the patients in developing countries present with LACC and are likely to gain from integration of MRI-based BT, the transition from standard to MRI-based BT is associated with distinct financial and logistic challenges, making it difficult for most of the busy departments in the developing world. In 2014, we decided to deliver increased doses to point A (> 80 to 84 Gy), compared with our historical doses of 70 to 75 Gy. X-ray (with occasional computed tomography [CT]/MRI)–based treatment planning was used. This article represents an audit of patients treated with a new regimen using a triaged image-based planning approach.

## METHODS

After obtaining institutional ethics committee approval, the clinical database of patients registered and treated outside of interventional clinical trials for LACCs in 2014 was reviewed. In addition, patients treated in 2015 within nonintervention trials were included. Those receiving postoperative or salvage radiation, those referred for BT alone from other institutions, and those receiving palliative treatment or neoadjuvant chemotherapy were excluded. Similarly, patients with retroviral disease were excluded.

### Treatment

Standard guidelines for management were followed. Following is the standard treatment policy we used during the study period. The prescribed treatment schedule included external radiation (46 Gy delivered in 23 fractions over 4.5 weeks) with weekly cisplatin (40 mg/m^2^). Those with deranged creatinine clearance (< 40 mL/min), anticipated poor tolerance because of age, or preexisting comorbidities received radiotherapy alone.

Radiation was delivered using the box field technique with conformal shielding. The cranial field boundary was the L4 to L5 junction, and the caudal border was defined 2.5 to 3.0 cm below the lowermost disease extent, ensuring adequate coverage of obturator lymph node regions. All pelvic-draining lymph node regions were included. Those with para-aortic lymphadenopathy received extended field radiation to a dose of 45 Gy/25#/5 weeks with concurrent cisplatin. In pelvic nodal positivity, the external radiation dose was individualized (46 or 50 Gy/23 to 25 fractions) on the basis of location of the involved node and anticipated contribution of dose to the node during intracavitary BT.

The planning aim was to deliver four fractions of high-dose-rate BT insertions of 7 Gy each to point A, to achieve a total dose of 80 to 84 Gy to point A, while restricting the International Commission on Radiation Units (ICRU) rectal point or rectal 2 cm^3^ and ICRU bladder point or bladder 2 cm^3^ doses to < 75 Gy EQD2 Gy_3_ and < 90 Gy EQD2 Gy_3_, respectively. The type of BT was often decided by response to external radiation during examination under anesthesia. Patients with favorable response underwent intracavitary BT with either tandem ovoid or tandem ring application, and those with significant residual disease beyond medial parametrium received intracavitary with additional interstitial needles using the Vienna applicator. During BT, procedure ultrasonography was used to confirm tandem placement within the uterine cavity.

Patients either underwent orthogonal x-ray– or CT-assisted treatment planning for BT depending on physician preference or availability of imaging resources within the busy department. ICRU bladder point and ICRU rectal and additional dose points were defined on orthogonal x-rays while organ at risk (rectum/bladder) contouring was performed on CT imaging. With a planning aim of 7 Gy to point A every fraction, the treatment plan was optimized to maintain the ICRU rectal and ICRU bladder dose constraints or the 2 cm^3^ rectal and bladder dose constraints.

Those with significant residual disease and additional interstitial needles underwent CT- or MRI-based BT planning. Here, the prescription was to high-risk clinical target volume (HRCTV). HRCTV delineation was performed using standard recommendation for those undergoing MRI-based planning.^6,7^ In those undergoing CT-based planning, the prescription target was decided based on examination under anesthesia, and the target was implanted accordingly.

Compliance with treatment was ensured by treatment audits. After completion of planned treatment, all patients underwent routine follow-up during the 6- to 8-week post-treatment period, every 3 to 4 months for the next 2 years and every 6 months thereafter. Follow-up evaluation involved clinical examination, including gynecologic examination and evaluation of rectal, bladder, and gastrointestinal toxicities. Sexual rehabilitation and vaginal dilatation exercises were advised. Follow-up imaging was performed based on symptoms and histologic confirmation obtained whenever feasible. Patients not attending routine follow-up were telephonically contacted to report for the follow-up.

### Data Collection

Clinical, treatment, and follow-up data for patients were retrieved from the electronic medical records and radiation oncology information system. Specifically, variables such as age, comorbidities, and International Federation of Gynecology and Obstetrics stage at presentation were recorded. Treatment data, such as prescribed radiation and BT dose, were obtained. Cumulative EQD2 to point A, HRCTV (wherever applicable), the rectum, and the bladder was obtained using commercially available EQD2 sheets.^8^

Details regarding chemotherapy delivery, compliance, and response to treatment were obtained. Follow-up information related to local, nodal, para-aortic, and distant relapse was obtained.

### Statistical Analysis

Descriptive statistics, including proportion and median, were used to describe baseline parameters and clinical stage, EQD2, and rectal and bladder dose. Chemotherapy compliance was categorized as the number of chemotherapy cycles received (yes/no, less than four cycles, and four or more cycles). Any death or local, regional, or distant relapse (first event) was calculated as an event toward DFS calculation. All disease-related end points, such as local control and DFS, were estimated from the date of diagnosis to the time of the first event. Univariable and multivariable analyses were performed to test the impact of various patients and treatment-related factors on locoregional control and disease-free and overall survival. All statistical analysis was performed with SPSS version 21 software (SPSS, Chicago, IL).

## RESULTS

In 2014, 854 patients with cervical cancer were registered in the radiation oncology unit, 493 of whom were considered for treatment in the institute. Of the 493 patients, 273 were excluded for various reasons, including (1) patients treated within interventional clinical trials (n = 132); (2) those receiving postoperative radiation (n = 62); (3) those with retroviral disease (n = 14); (4) those who received neoadjuvant treatment (n = 16); (5) those who received external radiation outside our institute; and (6) those who received only BT at our institute (n = 49). A total of 220 patients in 2014 were included for the analysis. After exclusion, a total of 220 patients in year 2014 were eligible. Another 136 patients treated within noninterventional trials in year 2015 were also included, resulting in a total of 356 patients for the final database. Of these, three received incomplete treatment, and 14 were lost to follow-up after completing treatment, leaving 339 evaluable patients for the present analysis.

**Fig 1:**
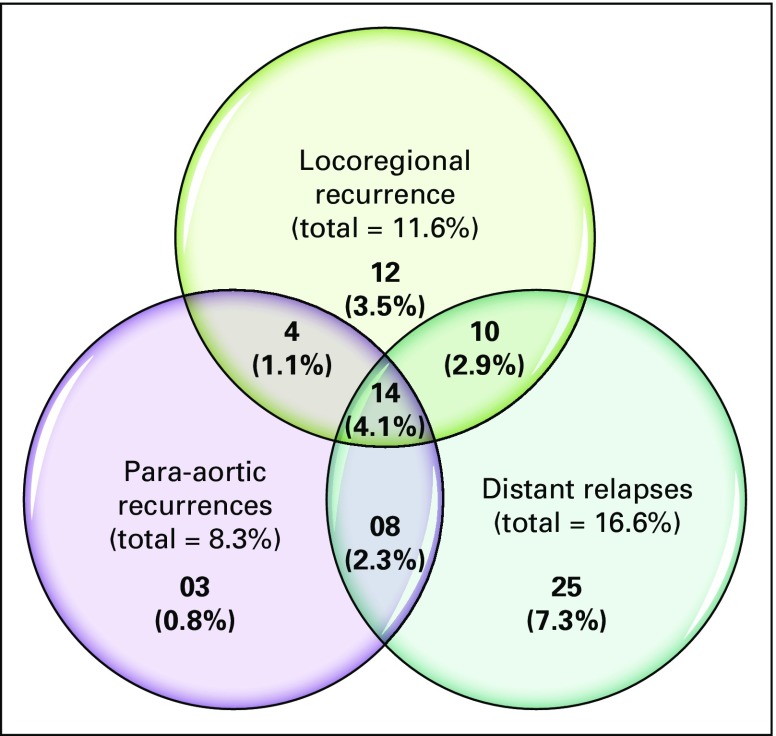
Venn diagram depicting patterns of relapse.

The treatment characteristics of the patients are listed in Table 1. The median age of the study cohort was 52 (32 to 81) years. Overall, 52% of patients had stage IB2 to IIB disease, and 48% had stage III to IVA. Overall, 23.3% (79 patients) had gross nodal disease at presentation. The majority of patients received pelvic radiotherapy, whereas 12 patients with para-aortic lymph nodes (3.5%) received extended field radiation with concurrent chemotherapy. A total of 289 of the 339 patients (85%) received concurrent CRT, whereas others received radiotherapy alone (Fig 1). The most common reasons for chemotherapy omission were related to advanced age (n = 16), anticipated poor tolerance because of multiple comorbidities (n = 5), and decreased creatinine clearance (n = 23). Reasons for prescribing radiation alone were not clearly documented for six patients. Of the 289 patients proceeding to receive concurrent chemotherapy, 87% received four or more cycles. A total of 19 patients received only one to two cycles because of worsening renal function (n = 12), grade III diarrhea (n = 3), or grade ≥ II hematologic toxicity. Other patients receiving up to three cycles had fewer cycles either because of anemia requiring correction or delay in administration of chemotherapy due to resolving toxicity (Table 2).

**Table 1:**
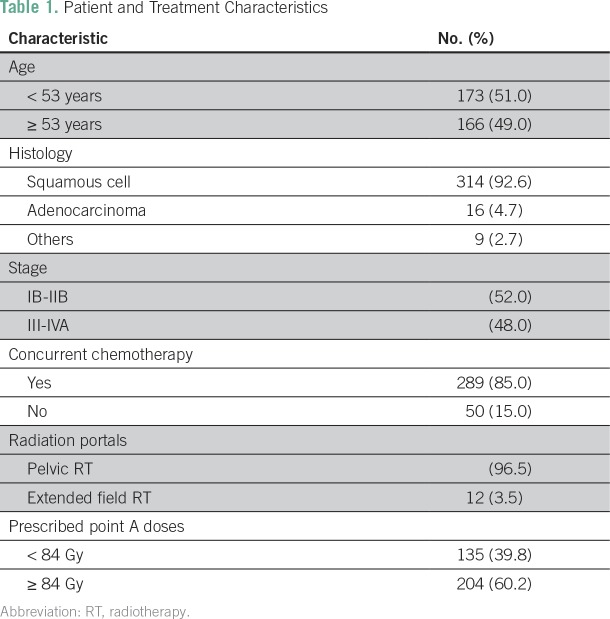
Patient and Treatment Characteristics

**Table 2:**
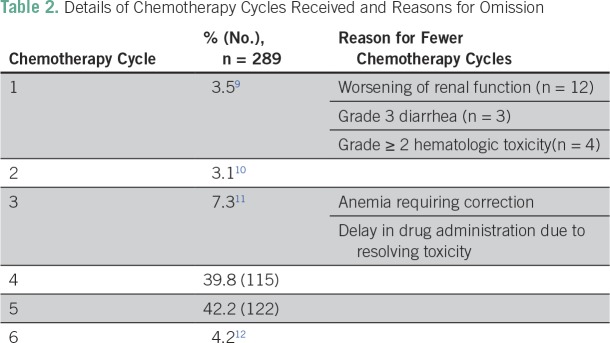
Details of Chemotherapy Cycles Received and Reasons for Omission

### Brachytherapy

Of the 339 patients, the majority (95%) had standard tandem-ovoid– or tandem-ring–based BT. The Vienna applicator with interstitial needles was used in 5% of patients (n = 17). Point A–based treatment planning was used in 95% of patients. The choice of BT imaging was the decision of the treating clinician in patients with significant response to external radiation. Of those undergoing Vienna applicator–based intracavitary and interstitial needles, 3.5% (12 patients) underwent CT-based planning, whereas 1.5% (n = 5) received MRI-based planning. Using this approach, the planned median dose to point A was 84 Gy (64.8 to 89.7 Gy). Overall, 17% of patients received EQD2 of < 80 Gy to point A, and in this cohort, the median EQD2 was 76 Gy. The median rectal and bladder doses were 73.5 Gy_3_ (interquartile range, 69.6 to 78.4 Gy) and 83 Gy_3_ (interquartile range, 73.2 to 90 Gy). The median overall treatment time (external-beam radiation therapy plus BT) was 9 weeks (range, 61 to 72 days).

### Disease Outcomes

**Fig 2:**
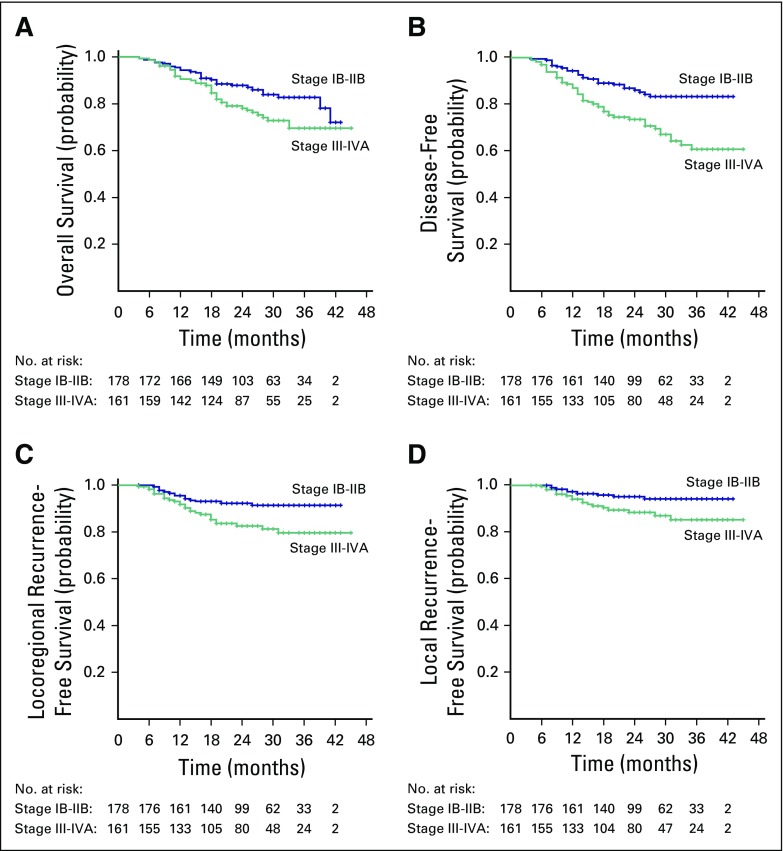
Figure depicting survival outcomes for stage IB2 to IIB and stage IIB to IVA disease: (A) overall survival, (B) disease-free survival, (C) locoregional recurrence-free survival, and (D) local recurrence-free survival.

At a median follow-up of 28 months (range, 4 to 45 months), 76 patients (22.4%) had relapsed. Of these, 57 (75%) had distant relapses, with the most common sites being liver and lung. Overall, 40 of the 76 patients (52.6%) had locoregional relapse, with 27 patients (35.5%) experiencing local recurrence. Pelvic nodal relapses were observed in 20 patients (26.3%). Para-aortic recurrence occurred in 29 patients (35.2%), and isolated para-aortic relapse occurred in three patients. Of all the para-aortic recurrences, two patients had documented para-aortic disease at presentation. The patterns of relapse are depicted in Figure 2.

The 3-year local control, locoregional control, DFS, and overall survival rates for stage IB to IIB disease were 94.1%, 91.3%, 83.3%, and 82.7%, respectively. The corresponding rates for stage III to IVA disease were 85.1%, 79.5%, 60.7%, and 69.6%, respectively. Local relapse rates were similar in patients whose doses were > 80 Gy_10_ or < 80 Gy_10_ to point A. Similarly, no difference was observed with doses > 84 Gy_10_ or < 84 Gy_10_ to point A.

Table 3 depicts the univariable and multivariable analyses evaluating various prognostic factors affecting the outcomes. Stage at presentation, para-aortic lymphadenopathy at presentation, use of concurrent chemotherapy, administration of four or more cycles of concurrent cisplatin, hydroureteronephrosis at diagnosis, and pretreatment albumin level (nutritional status) significantly affected the overall survival, whereas stage at presentation and age only affected DFS on univariable analysis. However, only low pretreatment albumin level and para-aortic lymph node enlargement independently affected overall survival on multivariable analyses (*P* = .038 and .010, respectively).

**Table 3:**
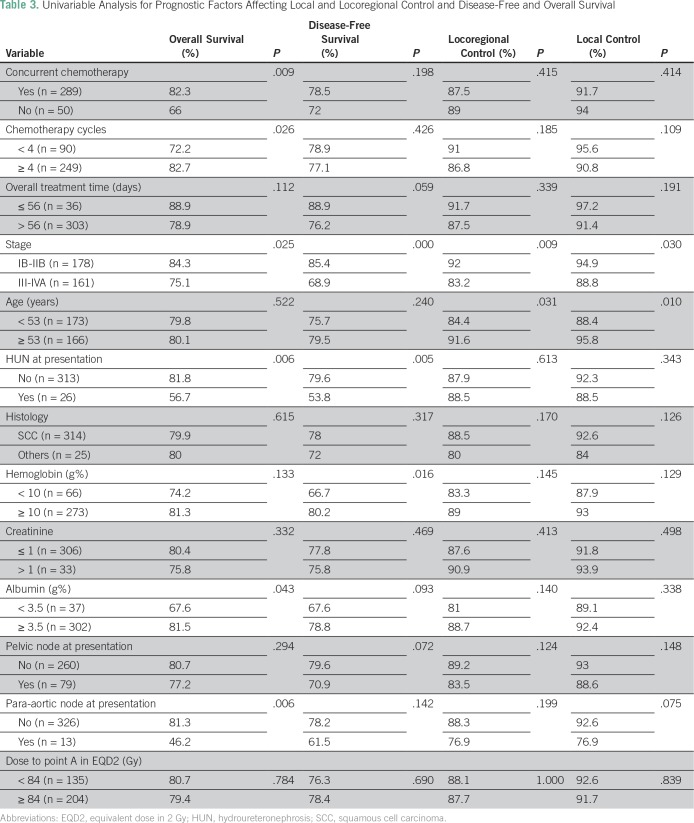
Univariable Analysis for Prognostic Factors Affecting Local and Locoregional Control and Disease-Free and Overall Survival

### Late Toxicity

At a median follow-up of 28 months, grade II, III, and IV proctitis and cystitis were observed in 14.5%, 4.4%, and 0.3%, and 2.9%, 0%, and 0% of patients, respectively. It is also noteworthy that 15 patients who developed grade III proctitis were graded as such because of a requirement for blood transfusions.

The median doses received by 2 cc rectal volume (D2cc) was 73.5 Gy, whereas the median dose was 73.4 Gy EQD2 in 10 patients who developed grade I toxicity, the median was 75 Gy EQD2 doses in 49 patients who developed grade II toxicities, and the median was 73.5 Gy EQD2 in 15 patients who developed grade III toxicities. Two patients (0.6%) developed rectovaginal fistula, for whom diversion sigmoid stoma was performed, and one patient died as a result of intestinal perforation at a median follow-up of 31 months (mean, 76 Gy). The median dose to ICRU bladder point/2 mL doses was 82 Gy. Dose received by 2cc bladder volume (D2cc) in 10 patients who developed grade II bladder toxicity were 82 Gy EQD2.

## DISCUSSION

Dose-escalated schedules have been used in the past decade in centers in Europe and essentially within the setting of MRI-based BT, wherein the prescription is to HRCTV rather than point A. Dimopoulos et at^9^ and Mazeron et al^10^ demonstrated improved local control with MRI-based BT, prescribing doses greater than 85 Gy to HRCTV. The dose received by 90% of the target (D90) for HRCTV > 87 Gy resulted in a local relapse incidence of 4% compared with 20% for D90 < 87 Gy. Mazeron et al^10^ demonstrated that total doses > 89 Gy and > 97 Gy needed to be reached to HRCTV in stage IIB and IIIB, respectively, to achieve a 90% probability of local control. However, the proposed dose escalation algorithms were within the setting of MRI-based target-concept and target-adapted BT.

A recent comparison of dose-escalated MRI-based BT performed within the EMBRACE study cohort at our center demonstrates improvement in outcomes compared with a historical cohort.^12,13^ More recently, the ICRU 89 and European Society for Medical Oncology-European Society for Radiotherapy and Oncology-European Society of Pathology guidelines have also been changed to recommend the use of image-adaptive BT, preferably with integration of MRI during BT.^14,15^ However, because integration of MRI-based BT has its own distinct logistic challenges, the transition has been slow, even in developed countries, with only up to 40% using MRI-based BT.^16-19^

Because the majority of patients with LACC are in the developing world, it is likely that transition to advanced BT techniques may be even slower than in the Western world. With such high disease burden, it is challenging to systematically implement MRI-based BT and maintain overall treatment time in all patients. Ours is a tertiary care center that has access to advanced BT applicators and MRI scanners; however, the clinical case load for > 2,500 BT applications for cervical cancer per year and six to eight BT applications per day makes it difficult to implement MRI-based BT for all patients, often compromising overall treatment time.

Although volume-based prescription with integration of MRI has been proposed to facilitate high BT doses to the target, it has not been proven whether similar local control can be achieved if doses to point A are escalated in the absence of MRI. Because outcomes of LACC using a dose-escalated regimen are not widely reported outside the setting of MRI-based BT, the results from this institutional audit represent a large cohort treated with this regimen to point A outside an interventional clinical trial. Because our dose prescription was to point A rather than HRCTV, it is likely that HRCTV, which was hitherto not defined in our patient population, would have received > 90 Gy in several complete responders. The 3-year local control of 94.1% and 85.1% in stages IB to IIB and III to IVA, respectively, is better than our previously reported historical cohort and comparable to our recently published experience of MRI-based BT and Retro-EMBRACE,^12,13,20^ suggesting that the benefit of dose deposited with regard to tumor control is not completely dependent on the imaging used for target delineation.

Although most of the studies integrating advanced BT emphasize improving local control with the goal of improving DFS, careful evaluation demonstrates a high rate of distant metastasis in stage IIIB to IVA tumors, thereby reducing the overall impact of gain in local control through advanced image guidance.^4^ Gains in overall outcomes can also be achieved by better integration and delivery of concurrent chemotherapy while using a dose-escalated regimen. In our cohort, 85% of patients were eligible to receive concurrent chemotherapy, and of these, only 87% could receive four or more cycles of concurrent chemotherapy. Although we could not demonstrate the impact of this deviation on local control, a trend toward improved survival was noted in women receiving more than four cycles of cisplatin. Therefore, adequate care needs to be taken to improve concurrent chemotherapy compliance.

The grade II and III toxicity observed within our cohort is marginally higher than that reported in the EMBRACE study (grade II rectal, 14.4%, compared with 11%), and could possibly be attributed to use of 2D planning in the majority of patients. Another group of 60 patients with LACC treated in our institution using an ultrasound- and CT-based approach for delineation of clinical target volume and prescription demonstrated similar local control with reduced toxicity at a median follow-up of 37 months.^21^ Since 2016, to further reduce treatment-related morbidity, we have initiated transition to ultrasound-guided placements and CT-based planning for all patients at first fraction, with MRI-based planning restricted to poor responders. We believe that this approach will further optimize local control and minimize late effects.

Although ICRU 89 and European Society for Medical Oncology-European Society for Radiotherapy and Oncology-European Society of Pathology guidelines recommend image-guided adaptive BT, preferably with MRI, for all patients, the results of this study suggest that comparable results can be achieved with triaged imaging and an implant approach. This may be a practical approach for both developed countries facing logistic challenges with MRI integration and developing countries that face financial challenges in implementing BT for cervical cancer. In a busy clinical department, it is often difficult to adhere to an 8-week treatment regimen with four BT fractions in LACC. Although a recently reported phase III International Atomic Energy Association trial demonstrated superiority of 7 Gy × 4 fractions over 9 Gy × 2 fractions for cervical cancer BT, there is a need to abbreviate BT schedules to equieffective lesser fractions (eg, 8 Gy × 3 fractions) to improve compliance with overall treatment time.^11^

The results of this audit are encouraging, where our 3-year outcomes are comparable to patients treated with more advanced techniques within clinical trials, albeit with some increase in treatment-related late toxicity. Further improvement in outcomes should be feasible by improving compliance with overall treatment time and chemotherapy, and by using CT-based BT or hybrid BT,^22,23^ thereby improving dose reporting to organs at risk.

The results of this audit demonstrate that point A–based BT prescription regimens can be safely implemented with careful reduction of organ-at-risk doses whenever indicated. In the absence of level-I evidence demonstrating incremental benefits of MRI-based BT, a triaged imaging and dose prescription approach could present an equieffective alternate strategy.
